# Examination of factors impacting spitting or vomiting among children under 5 years of age during seasonal malaria chemoprevention: a quantitative study in Burkina Faso, Chad, Nigeria and Togo

**DOI:** 10.1186/s41182-024-00642-z

**Published:** 2024-11-12

**Authors:** Chen Gao, Sikai Huang, Taiwo Ibinaiye, Benoît Sawadogo, Adama Traore, Cheick Saïd Compaoré, Fantche Awokou, Chukwudi A. Nnaji, Kevin Baker, Duoquan Wang, Sol Richardson

**Affiliations:** 1 Department of Epidemiology and Biostatistics, School of Public Health, Anhui Medical University, Hefei, 230000 Anhui China; 2 Vanke School of Public Health, Tsinghua University, Beijing, 100084 China; 3 Malaria Consortium Nigeria, 33 Pope John Paul Street, Maitama, Abuja-FCT, Nigeria; 4 Malaria Consortium Burkina Faso, Secteur 24, Rue 30.81 Arrondissement 5, 1er Etage, Immeuble ADIZ, 06 BP 9519 Ouagadougou 06, Burkina Faso; 5 Malaria Consortium Togo, Malaria Consortium, Programme National de Lutte Contre Le Paludisme (PNLP) Quartier Administratif, Rue Adamé, Lomé, Togo; 6 Malaria Consortium London, The Green House, 244-254 Cambridge Heath Road, London, E2 9DA UK; 7 Department of Global Public Health, Karolinska Institute, Stockholm, Sweden; 8 Chinese Center for Disease Control and Prevention, National Institute of Parasitic Diseases, Shanghai, China; 9 Chinese Center for Tropical Diseases Research, Shanghai, China; 10 WHO Collaborating Centre for Tropical Diseases, Shanghai, China; 11 National Center for International Research on Tropical Diseases, Ministry of Science and Technology, Shanghai, China; 12 Key Laboratory of Parasite and Vector Biology, Ministry of Health, Shanghai, 200025 China

**Keywords:** Malaria, Cross-sectional survey, Child health, Chemoprophylaxis

## Abstract

**Background:**

Since 2012, the World Health Organization has recommended seasonal malaria chemoprevention (SMC) with sulfadoxine-pyrimethamine plus amodiaquine (SPAQ) for children aged 3–⁠59 months in regions where malaria transmission is seasonal. Full ingestion of SMC medicines without spitting or vomiting during a complete 3-day course is critical to ensure effectiveness of SMC medicines and to avoid development of antimalarial resistance. Although evidence suggests that spitting or vomiting is not rare, there is limited analytical evidence on potential factors associated with spitting or vomiting in SMC campaigns.

**Methods:**

We utilized data from SMC coverage surveys conducted in Burkina Faso, Chad, Togo and Nigeria between 2020 and 2022. Episodes of spitting or vomiting were defined as SMC-eligible children spitting out most of the dose or vomiting the entire dose within 30 min of SPAQ administration as reported by primary caregivers. We conducted a cross-sectional study using mixed-effects logistic regression with variables including household socioeconomic variables and caregiver knowledge of SMC, to identify factors associated with spitting or vomiting.

**Results:**

The proportion of SMC-eligible children spitting or vomiting SPAQ doses ranged from 1.81% in Nigeria to 4.36% in Chad. The odds of spitting or vomiting were lower among children administered medicines under community distributor (CD) supervision, and whose primary caregivers had a high degree of knowledge of SMC. Spitting or vomiting were negatively associated with caregiver adherence to AQ administration and caregiver reporting of children’s adverse reactions to SMC medicines. Over half of the children experiencing a spitting or vomiting episode did not receive a replacement dose from CDs. Redosing was positively associated with caregiver educational attainment, caregiver knowledge of SMC, and directly supervised medicine administration.

**Conclusions:**

CD-supervised administration of SPAQ can strengthen community engagement strategies to enhance appropriate administration and full ingestion of SMC medicines according to the SMC delivery protocol.

## Background

Malaria remains one of the leading causes of morbidity and mortality in sub-Saharan Africa. Globally, in 2022, there were an estimated 249 million malaria cases in 85 malaria-endemic countries and areas, with an estimated 608,000 malaria deaths [1]. The proportion of children aged under 5 years among total malaria cases was 36.6%, but children aged under 5 years, representing up to 73% of deaths related to malaria, are the most vulnerable group [1, 2]. Global efforts, including vector control, preventive chemotherapies, malaria vaccines, and appropriate case management, have been deployed to protect children from malaria infection [2]. In 2012, the WHO recommended introduction of seasonal malaria chemoprevention (SMC) to prevent malaria morbidity and mortality in children aged under 3–59 months in areas of highly seasonal malaria transmission in sub-Saharan Africa [3]. In 2022, SMC was implemented in 17 sub-Saharan African countries, reaching an estimated 49 million eligible children [4, 5]. Accumulated evidence from randomized control trials and large-scale implementation of SMC indicates that SMC is safe and effective among targeted eligible children, with a preventive effectiveness against malaria cases ranging from 73 to 98% [6–8]. SMC with sulfadoxine-pyrimethamine (SP) and amodiaquine (AQ), administered at 28-day intervals to all children under 5 years of age during each malaria transmission season, has been associated with a 75% reduction in incidence of all malaria episodes and severe malaria episodes, and a reduction in child mortality from 3 per 1000 per year to 2 per 1000 per year [9–11].

SMC involves intermittent administration of a complete course of SPAQ to prevent malaria cases by maintaining therapeutic drug concentrations in the blood during the high transmission season [5, 12]. Before the Coronavirus (COVID-19) pandemic, SMC community distributors (CDs) typically provided a blister pack of SPAQ for a complete 3-day course. This typically occurred via door-to-door household visits, with direct administration of SPAQ to eligible children on Day 1 by CDs. Subsequently, caregivers of eligible children are instructed to administer the remaining two daily doses of AQ on both Day 2 and Day 3 without CD supervision. However, during the COVID-19 pandemic, the SMC delivery protocol was adapted to minimize contact with beneficiaries. CDs were requested to supervise caregivers when they administered the first dose of SP and AQ to their children on Day 1 at a safe distance [13]. A 3-day complete administration of SMC medicines and the following 28-day protective period define a SMC cycle, of which there are typically four to five in an annual SMC round [11].

Several randomized trials have suggested that combination therapy preventive strategies using SPAQ outperform other combination therapies (e.g., SP + artesunate, SP + AQ + artesunate) and found no severe adverse reactions. However, mild to moderate adverse reactions have been linked with AQ administration, with vomiting and headache being the most common [14, 15]. Due in part to the bitter unpleasant taste of AQ, children often exhibit varying degrees of spitting or vomiting immediately following administration of SMC medicines [16]. Failure to fully ingest all doses may undermine the protective effect of SMC against malaria. A monitoring and evaluation study in Burkina Faso reported that 38% of suboptimal administration of SMC medicines was attributable to children’s adverse reactions to SMC medicines, with vomiting accounting for approximately half of these adverse reactions [17]. A descriptive study in Burkina Faso, Mali and Niger identified vomiting as the second major cause of non-adherence to administration of the second and third doses of AQ [18]. Moreover, another study in Nigeria, Burkina Faso, Chad, and Togo based on data from routine end-of-round SMC coverage surveys found that previous adverse events following administration of SMC medicines to eligible children were associated with reduced caregiver adherence to Day 2 and Day 3 AQ administration [19]. The WHO recommends administration of a replacement dose after allowing the child to rest for about 10 min if a child spits or vomits within 30 min during directly supervised administration of Day 1 SPAQ [5]; redosing aims to ensure that children receive the correct medicine dose to decrease the impact of non-adherence on resistance and provide long-lasting protection during the malaria transmission season [5].

A cross-sectional study conducted by Malaria Consortium found that some caregivers refused to administer SMC medicines to their children or did not administer them correctly during the COVID-19 pandemic [20, 21]. This may exacerbate spitting or vomiting at the time of administration of SMC medicines. Targeted interventions have been developed to promote caregiver compliance to correct administration amid the pandemic, in addition to delivery of key messages via CDs. In Nigeria, the lead mothers intervention, delivered by female residents in SMC settings aged 18 years and above who visit caregivers in their homes and disseminate messages following door-to-door visits by CDs, has been proven to be effective at promoting caregiver adherence to AQ administration [19]. In Burkina Faso, Chad, and Togo, the role model approach, a community-driven behavior change strategy, has been piloted to explore its potential to address the challenges associated with SPAQ administration including spitting or vomiting [22].

Several recent studies have highlighted the frequency of vomiting and its impact on non-adherence to AQ administration in various sub-Saharan African countries. However, analysis of spitting or vomiting has largely remained at the summary statistics level, nesting within studies focusing on coverage and cost-effectiveness of SMC rather than specifically analyzing factors that facilitate or prevent spitting or vomiting of SMC medicines. Besides, recent updates to WHO guidelines for SMC emphasize monitoring and evaluation, and pharmacokinetic considerations, at the time of scaling up SMC programs or introducing new chemoprevention medicines [5], highlighting the need to improve understanding of relatively common spitting or vomiting events and potential measures to reduce their incidence.

This study aimed to examine factors influencing spitting or vomiting following receipt of SPAQ on Day 1 during the last cycle of household visits by CDs in four Sahelian countries (Burkina Faso, Chad, Nigeria, and Togo), using data obtained from annual routine end-of-round SMC coverage surveys commissioned by Malaria Consortium. Additionally, this study sought to assess redosing behavior and its associations with factors including characteristics of the selected child, caregiver, head of household and household, in addition to caregiver awareness and knowledge of SMC.

## Materials and methods

### Study location

This study was conducted using data collected in four sub-Saharan African countries including Burkina Faso, Chad, Nigeria, and Togo. SMC end-of-round coverage surveys were conducted in these four countries between 2020 and 2022 (except for Burkina Faso in 2021), spanning three annual rounds of SMC. Malaria Consortium’s SMC program has been recognized as a major contributor to malaria control and elimination across these four countries [23, 24]. In Nigeria, Malaria Consortium supported delivery of SMC in a total of nine eligible states in 2022, targeting a population of 10.72 million eligible children. Meanwhile, in Burkina Faso, SMC supported by Malaria Consortium covered 29 districts across 6 regions, reaching 2.11 million eligible children [18]. In Chad and Togo, 1.2 million from 27 districts and 0.51 million eligible children from 19 districts were reached by Malaria Consortium-supported SMC campaigns, respectively^18^. End-of-round surveys, commissioned by Malaria Consortium, were conducted by external contractors not involved in SMC implementation.

### Survey design

In all four countries, end-of-round surveys employed multi-stage random sampling combined with sampling of clusters by probability proportional to population size to select settlements at random to achieve a self-weighting sample [23]. In Nigeria, a modified multi-stage random sampling procedure was followed to select compounds, settlements, health facility catchment areas, Local Government Areas (LGAs), and states at random. Only households with at least one child aged 3–59 months were eligible for inclusion in end-of-round surveys. More detailed information on end-of-round survey protocols can be found elsewhere [19, 24, 25].

**Fig. 1:**
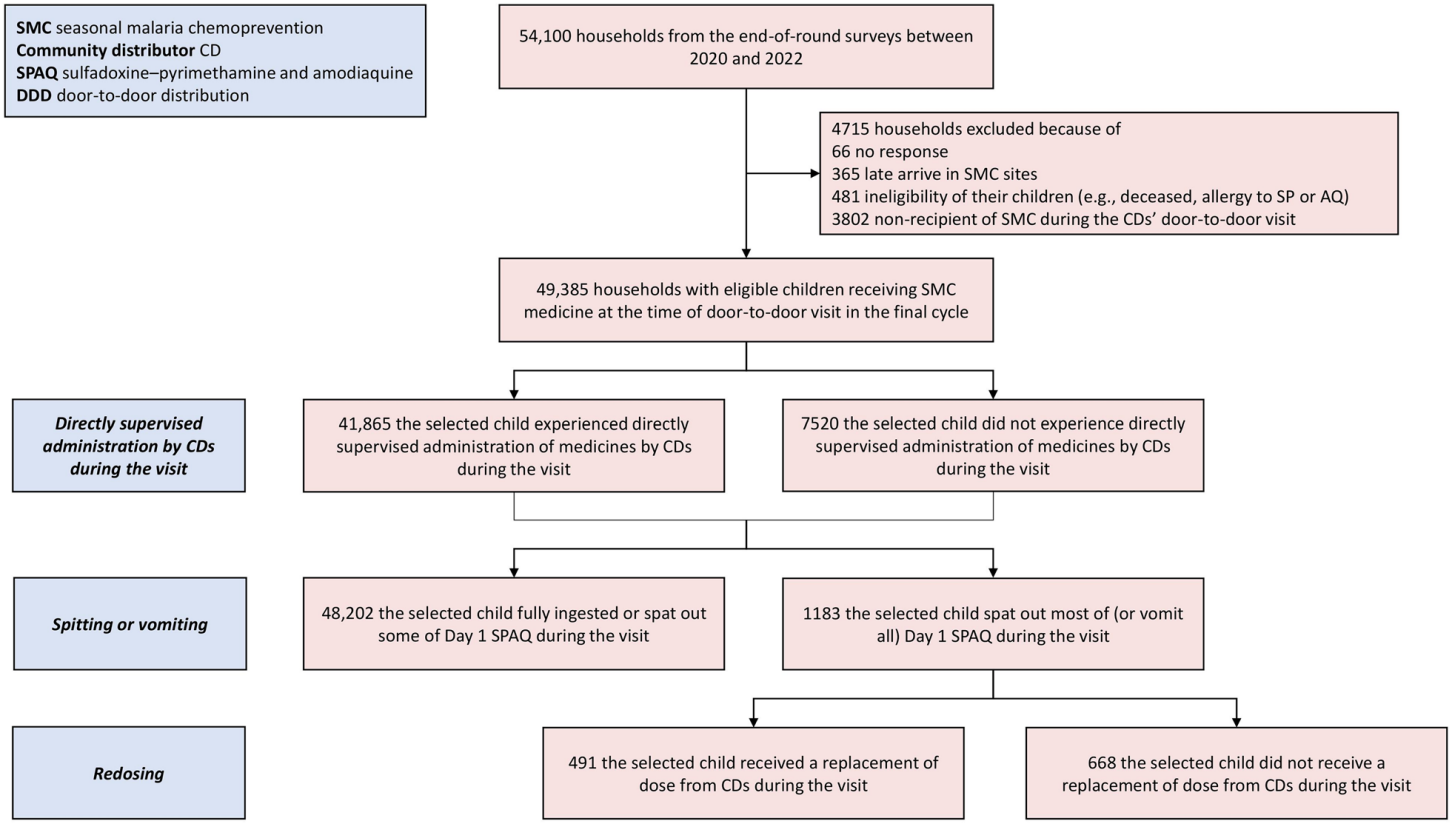
Flow chart of the study population

The study sample consisted of 54,100 households surveyed during end-of-round surveys. Households were excluded if: (1) they did not respond to the survey; (2) they arrived in the local community after initiation of the final SMC cycle; (3) the selected child did not receive SMC medicines at the time of door-to-door household visits by CDs in the final SMC cycle of that year; or (4) the selected child was not at home or ineligible for other reasons (e.g., deceased, allergy to SMC medicines, sick) during household visits [5]. After excluding 4715 households, the final analytic sample comprised 49,385 households (Fig. 1).

### Household interviews

Pairs of data collectors visited each household door-to-door and conducted computer-assisted personal interviews using the application Survey CTO version 2.70 on mobile devices. Survey CTO randomly selected one eligible child from the household from a roster, and all survey questions related to that child, their primary caregiver, head of household, and household. A primary caregiver in this survey referred to any individual, aged 15 years or over, with the primary responsibility for the feeding and daily care of at least one child under the age of 5 and a household head referred to a member of the family who manages the resources and is the final decision maker in the household, as reported by household members. Data on a range of variables were available for analysis, including socioeconomic and demographic characteristics of selected household members, key indicators on SMC coverage and adherence, and outcomes of SMC administration. Interviews were conducted in local languages using questionnaires provided by Malaria Consortium, with data collectors translating on the spot from English or French and assigning responses to predefined answer categories. Informed consent was obtained from all respondents prior to interviews.

### Variables

The outcome, spitting or vomiting, was defined as the eligible child spitting out most or almost all of the dose, or vomiting all of the dose, at the time of administration of Day 1 SPAQ during the final cycle of SMC in a given survey year. This was operationalized as a binary variable (yes/no), where "yes" indicated that the eligible child spat out or vomited most of or all of the dose of SMC medicines, and "no" indicated that the eligible child ingested the entire dose or only spat out some of the SMC medicine dose.

Potential predictors of spitting or vomiting considered for inclusion in the analysis included direct CD-supervised medicine administration (yes/no), child’s sex (female/male), child’s age (years), primary caregiver’s sex (female/male), caregiver’s age (under 29 years/30–39 years/40–49 years/50 and above), caregiver’s self-reported literacy (yes/no), caregiver’s highest level of education (none or informal/primary/secondary and higher), caregiver’s employment status (yes/no), caregiver’s knowledge and awareness of SMC, head of household’s self-reported literacy (yes/no), head of household’s highest level of education (none or informal/primary/secondary and higher), head of household’s employment status (yes/no), and household socioeconomic status. Caregiver awareness and knowledge of SMC was operationalized as an ordinal variable (low/medium/high), based on spontaneous responses to six questions relating to SMC (categorized according to whether they were correct, yes/no) including awareness of the purpose of SMC; awareness of criteria for children’s eligibility to SMC; knowledge of the importance of administering SMC medicines only children aged under 5 years; knowledge of the reason for administration of Day 2 and Day 3 AQ; knowledge of appropriate reporting of adverse reactions following receipt of SMC medicines; and belief in the effectiveness of SMC at providing protection against malaria. Caregivers were considered to have a low level of knowledge and awareness of SMC if they spontaneously responded with the correct answer (or similar response) to at most two questions, medium level if they answered 3–5 questions correctly, and high level if they answered all questions correctly. Household socioeconomic status was assessed using the latest country-specific Simple Poverty Scorecards and operationalized as a binary variable for household poverty status (defined as > 70% probability household is below the poverty threshold of 1.90 purchasing power parity [PPP]-adjusted 2011 United States Dollars [USD] per day, using the 2014/2015 definition) [26–29]. In households where the primary caregiver and head of household were the same person, observations for both variables were the same.

Furthermore, we introduced a secondary outcome, redosing, defined as children receiving a replacement dose of SMC medicines from CDs immediately after spitting or vomiting at the time of administration of Day 1 SPAQ, to examine the patterns of redosing behavior. This was based on caregiver self-reporting and operationalized as a binary variable (yes/no). Assessed potential predictors of redosing included full adherence to administration of both the second and third dose of AQ (yes/no), occurrence of adverse reactions to either SPAQ on Day 1 or AQ on Day 2 or Day 3 excluding vomiting in the selected eligible child (yes/no), caregiver reporting of their adverse reactions to CDs or health facility personnel (yes/no), and lead mothers visit during the previous SMC cycle (in Nigeria only, yes/no).

### Statistical analysis

Post-sampling survey weights were generated for country sample size versus population to account for survey design and correct for over- or underrepresentation of survey respondents due to differences in country sample sizes; these were applied throughout this analysis (Table 1).

**Table 1:** Summary of sample characteristics by country

Variable (Total *n* = 49,385)	Burkina Faso	Chad	Nigeria	Togo	Total
	*n* (%)^a^	*n* (%)^a^	*n* (%)^a^	*n* (%)^a^	*n* (%)^a^
Survey year					
2020	2139 (43.52)	2285 (29.72)	6361 (27.97)	1861 (32.50)	12,646 (29.69)
2021	0 (0.00)	2914 (33.29)	10,242 (34.76)	1884 (33.28)	15,040 (31.42)
2022	2896 (56.48)	3297 (36.99)	13,388 (37.27)	2118 (34.22)	21,699 (38.89)
Child					
Direct CD-supervised administration					
Yes	4473 (88.86)	6805 (79.77)	25,649 (84.48)	4938 (84.07)	41,865 (84.49)
No	562 (11.14)	1691 (20.23)	4342 (15.52)	925 (15.92)	7520 (15.51)
Spitting or vomiting					
Yes	130 (2.59)	360 (4.36)	508 (1.81)	185 (3.19)	1183 (2.13)
No	4905 (97.41)	8136 (95.64)	29,483 (98.19)	5678 (96.81)	48,202 (97.87)
Redosing (if child did not ingest all of the SMC medicine dose)					
Yes	60 (57.19)	150 (40.77)	221 (41.72)	60 (32.11)	491 (42.47)
No	46 (42.81)	210 (59.23)	287 (58.28)	125 (67.89)	668 (57.53)
Adherence to the second and third dose of AQ					
Yes	4974 (98.80)	8151 (95.87)	29,268 (97.30)	5744 (97.96)	48,137 (97.34)
No	61 (1.20)	345 (4.13)	723 (2.70)	119 (2.04)	1248 (2.66)
Sex					
Male	2518 (50.02)	4746 (55.84)	15,367 (50.96)	3080 (52.52)	25,711 (51.32)
Female	2517 (49.98)	3750 (44.16)	14,624 (49.04)	2783 (47.48)	23,674 (48.68)
Age					
3–11 months	441 (8.79)	695 (8.26)	1924 (5.99)	568 (9.67)	3628 (6.56)
1 year	873 (17.33)	1437 (16.84)	4752 (15.75)	956 (16.27)	8018 (16.00)
2 years	1063 (21.12)	2268 (26.63)	6552 (22.07)	1194 (20.35)	11,077 (22.28)
3 years	1061 (21.07)	2033 (23.89)	7010 (23.49)	1312 (22.43)	11,416 (23.26)
4 years	1276 (25.32)	1656 (19.51)	7474 (24.96)	1416 (24.14)	11,822 (24.53)
5 years	321 (6.37)	407 (4.86)	2279 (7.73)	417 (7.14)	3424 (7.36)
Adverse reactions to SMC medicines^b^ (*N* = 46,898)					
Yes	304 (6.24)	1130 (13.99)	2870 (9.91)	543 (9.87)	4847 (9.88)
No	4573 (93.76)	6825 (86.01)	27,121 (90.09)	4965 (90.13)	42,051 (90.12)
Primary caregiver					
Sex					
Male	539 (10.64)	2238 (26.32)	4723 (16.12)	860 (14.75)	8360 (16.38)
Female	4496 (89.36)	6258 (73.68)	25,268 (83.88)	5003 (85.25)	41,025 (83.62)
Age					
Under 29 years	2356 (46.88)	3800 (44.85)	14,134 (47.68)	2649 (45.10)	22,939 (47.29)
30–39 years	1993 (39.50)	3032 (35.69)	11,045 (35.99)	2351 (40.13)	18,421 (36.43)
40–49 years	490 (9.72)	1283 (15.00)	3599 (12.26)	624 (10.67)	5996 (12.19)
50 and more above	196 (3.90)	381 (4.47)	1213 (4.08)	239 (4.10)	2029 (4.09)
Self-reported literacy					
Yes	2057 (40.76)	4467 (52.27)	19,652 (64.74)	3089 (52.67)	29,265 (61.13)
No	2978 (59.24)	4029 (47.73)	10,339 (35.26)	2774 (47.33)	20,120 (38.87)
Level of highest education					
None or informal	3238 (64.36)	5423 (63.97)	15,799 (56.53)	2860 (48.78)	27,320 (57.55)
Primary	808 (16.02)	1444 (16.80)	4577 (15.44)	1371 (23.41)	8200 (15.89)
Secondary or higher	989 (19.62)	1629 (19.23)	9615 (28.03)	1632 (27.81)	13,865 (26.56)
Employment status					
Non-employed	1736 (34.40)	4516 (53.54)	10,198 (35.08)	1216 (20.85)	17,666 (35.97)
Employed	3299 (65.60)	3980 (46.46)	19,793 (64.92)	4647 (79.15)	31,719 (64.03)
Awareness and knowledge of SMC					
Low	397 (7.81)	630 (7.48)	5494 (17.82)	819 (13.90)	7340 (15.94)
Medium	1627 (32.33)	2058 (24.53)	8503 (28.78)	2166 (36.98)	14,354 (29.07)
High	3011 (59.86)	5808 (67.99)	15,994 (53.40)	2878 (49.12)	27,691 (54.99)
Caregiver reporting of adverse reactions to CDs or health facility personnel (*N* = 4846)					
Yes	181 (59.65)	604 (53.04)	1989 (71.10)	276 (51.16)	3050 (67.73)
No	123 (40.35)	526 (46.96)	881 (28.90)	266 (48.84)	1796 (32.27)
Head of household					
Self-reported literacy					
Yes	906 (38.54)	3861 (54.61)	22,949 (77.39)	3537 (60.30)	31,253 (73.33)
No	1444 (61.46)	2166 (45.39)	7041 (23.61)	2325 (39.70)	13,976 (26.67)
Level of highest education					
None or informal	1534 (65.29)	4171 (59.47)	12,896 (45.92)	1118 (24.57)	19,719 (47.14)
Primary	386 (16.42)	1148 (16.14)	3426 (10.99)	1338 (29.02)	6298 (12.16)
Secondary or higher	430 (18.39)	1708 (24.39)	13,668 (43.09)	2139 (46.41)	17,945 (40.70)
Employment status					
Not employed	491 (20.75)	3044 (43.40)	4530 (15.28)	546 (9.41)	8611 (17.30)
Employed	1859 (79.25)	3983 (56.60)	25,325 (84.72)	5316 (90.59)	36,483 (82.70)
Household					
Poverty					
Yes	421 (8.57)	3318 (37.24)	1367 (4.76)	793 (13.82)	5899 (8.01)
No	4614 (91.43)	5178 (62.76)	28,624 (95.24)	5070 (86.18)	43,486 (91.99)
Lead mother visit (Nigeria only, *n* = 29,991)					
Yes			20,780 (69.13)		20,780 (69.13)
No			9211 (30.87)		9211 (30.87)

SMC: seasonal malaria chemoprevention, AQ: amodiaquine, CD: community distributor

^a^Weighted proportion based on weighted χ2 test

^b^Adverse reactions to the SMC medicines excluded “vomiting”

^c^Respondents who answer "yes" to "adverse reactions to the SMC medicines" were eligible to proceed to this question

First, we calculated frequencies (*n*) and weighted percentages (%) to describe the distribution of respondents’ characteristics by their child’s spitting or vomiting status. Weighted chi-square tests were performed to assess the association between these variables and spitting or vomiting.

We performed random-effects logistic regression models to identify predictors of spitting or vomiting at the time of Day 1 SPAQ administration, with random intercepts for survey year, country, and administrative subdivision (LGAs in Nigeria, districts in other countries) to account for clustering. A forward stepwise regression approach based on Collett’s method was used to select potential predictors for inclusion in the final regression models [30]; variables were sequentially added based on Wald test results, and they were retained if they improved model fit (*p*-value < 0.05). Results were displayed as odds ratios (OR) with 95% confidence intervals (CI).

We also assessed the association between redosing and the variables mentioned above; summary statistics (n and %) were calculated and weighted chi-squared tests were performed to identify variables significantly associated with redosing. Data were analyzed using Stata 17.0.

## Results

### Characteristics of surveyed household members in Burkina Faso, Chad, Nigeria and Togo

A total of 49,385 children were included in the analytic sample across the 2020, 2021 and 2022 SMC end-of-round surveys in the four countries (5035 in Burkina Faso, 8496 in Chad, 29,991 in Nigeria, and 5863 in Togo). Table 1 presents the selected child-, caregiver-, head of household-, and household-level characteristics by country. The majority of primary caregivers (84.49%) reported directly supervised administration of Day 1 SPAQ during door-to-door household visits by CDs. The overall proportion of caregivers who reported adherence to Day 2 and Day 3 AQ administration to the selected child was 97.34%. The overall weighted proportion of primary caregivers from the four countries who reported spitting or vomiting by their child was 2.13%, ranging from 1.81% in Nigeria to 4.36% in Chad. Of caregivers who reported spitting or vomiting, only 42.47% reported redosing, ranging from 32.11% in Togo to 57.19% in Burkina Faso.

Regarding primary caregivers’ characteristics across the four countries, 83.62% surveyed were female, ranging from 73.68% in Chad to 89.36% in Burkina Faso. Caregiver ages of under 29 (47.29%) and 30–39 (36.43%) accounted for the largest weighted proportions. The proportion of surveyed caregivers reporting being literate varied by country, ranging from 40.76% in Burkina Faso to 64.74% in Nigeria. The overall proportions of caregivers by education attainment were 57.55% with no or only informal education, 15.89% with primary education, and 26.56% with secondary education or higher. Overall, 54.99% of caregivers surveyed were categorized as having a high level of awareness and knowledge of SMC; this varied by country, ranging from 49.09% in Togo to 67.99% in Chad. The majority of heads of household were in employment (82.70%), while around 10% of households were considered to be in poverty (> 70% probability of household income below 1.90 PPP-adjusted 2011 USD per day); proportions of households in poverty varied by country, ranging from 8.57% in Nigeria to 37.24% in Chad.

### Characteristics of the survey household members by spitting or vomiting

Table 2 presents the results of weighted chi-square tests for associations between child-, caregiver-, head of household-, and household-level characteristics, and spitting or vomiting, with F-statistics and *p*-values. Male children were significantly less likely to present spitting or vomiting at the time of Day 1 SPAQ administration (F = 4.50, degrees of freedom (df) = 1, *p* = 0.042), while those in younger age groups (e.g., 3–11 months) were also less likely to present spitting or vomiting (F = 5.33, df = 5, *p* = 0.003). Statistically significant associations were found between caregivers’ sex (F = 7.73, df = 1, *p* = 0.009), caregiver’s literacy (F = 4.27, df = 1, *p* = 0.047), caregiver’s employment status (F = 3.48, df = 1, *p* = 0.072), caregiver awareness and knowledge of SMC (F = 32.43, df = 2, *p* < 0.001), and spitting or vomiting by the selected child. Children who received Day 1 SPAQ under the supervision of CDs were significantly less likely to present spitting or vomiting (F = 95.45, df = 1, *p* < 0.001). In Nigeria, lead mothers visits were negatively associated with spitting or vomiting.

**Table 2:** Summary of sample characteristics by children’s spitting or vomiting status at the time of Day 1 SPAQ administration

Characteristics (*N* = 49,385)	Spitting or vomiting	No spitting or vomiting	F-statistic	*p*-value ^a^
	n (%)^b^	n (%)^b^		
Child				
Direct CD-supervised administration				
Yes	778 (64.65)	41,087 (84.93)	95.45	<0.001***
No	405 (35.35)	7115 (15.07)		
Sex				
Male	580 (45.39)	25,131 (51.45)	4.50	0.042*
Female	603 (54.61)	23,071 (48.55)		
Age				
3–11 months	90 (7.38)	3538 (6.54)	5.33	0.003**
1 year	244 (22.41)	7774 (15.86)		
2 years	290 (23.56)	10,787 (22.26)		
3 years	236 (19.23)	11,180 (23.35)		
4 years	250 (21.47)	11,572 (24.60)		
5 years	73 (5.95)	3351 (7.39)		
Sex				
Male	160 (12.35)	8200 (16.47)	7.73	0.009**
Female	1023 (87.65)	40,002 (83.53)		
Primary caregiver				
Age				
Under 29 years	580 (48.27)	22,359 (47.26)	0.22	0.829
30–39 years	436 (36.67)	17,985 (36.43)		
40–49 years	125 (11.10)	5871 (12.21)		
50 or more above	42 (3.96)	1987 (4.10)		
Self-reported literacy				
Yes	655 (55.28)	28,610 (61.26)	4.27	0.047*
No	528 (44.72)	19,592 (38.74)		
Level of highest education				
None or informal	636 (56.91)	26,684 (57.57)	0.85	0.419
Primary	173 (13.56)	8027 (15.94)		
Secondary or higher	374 (29.52)	13,491 (26.50)		
Employment status				
Non-employed	505 (41.84)	17,161 (35.84)	3.48	0.072
Employed	678 (58.16)	31,041 (64.16)		
Awareness and knowledge of SMC				
Low	290 (27.26)	7050 (15.70)	32.43	<0.001***
Medium	397 (35.61)	13,957 (28.92)		
High	486 (37.13)	27,195 (55.38)		
Head of household				
Self-reported literacy				
Yes	709 (69.87)	30,544 (73.40)	3.70	0.064
No	366 (30.13)	13,610 (26.60)		
Level of highest education				
None or informal	463 (46.34)	19,256 (47.16)	0.21	0.787
Primary	137 (11.46)	6161 (12.17)		
Secondary or higher	441 (42.21)	17,504 (40.67)		
Employment status				
Non-employed	255 (20.86)	8356 (17.23)	1.23	0.276
Employed	815 (79.14)	35,668 (82.77)		
Household				
Poverty				
Yes	172 (9.77)	5727 (7.97)	1.19	0.284
No	1011 (90.23)	42,475 (92.03)		

SMC: seasonal malaria chemoprevention, CD: community distributor

*p* < 0.05, **p < 0.01, ***p < 0.001

^a^Two-sided *p* value for χ2 test for difference in proportions

^b^Weighted proportion based on weighted χ2 test

### Predictors of spitting or vomiting at the time of Day 1 SPAQ administration

Table 3 presents the results of the random-effects logistic regression analysis of spitting or vomiting across the four countries following variable selection by a forward stepwise approach. Variables selected for inclusion in the final model were child’s sex, child’s age, caregiver’s self-reported literacy, caregiver’s level of education, caregiver’s employment status, caregiver’s awareness and knowledge of SMC, head of household’s employment status, and directly supervised Day 1 SPAQ administration by CDs. Results of the logistic regression model showed that children aged 1 year had higher odds of spitting or vomiting at the time of Day 1 SPAQ administration; odds were significantly higher compared with children aged 3–11 months (OR = 1.41, 95% CI 1.08 to 1.83). Such a statistically significant association was not found, compared with children aged 3–11 months, among children aged over 2 years despite their lower odds of spitting or vomiting. Male children had lower odds of spitting or vomiting compared with female children (OR = 0.78, 95% CI 0.64 to 0.95). Meanwhile, caregiver self-reported literacy (OR = 0.88, 95% CI 0.86 to 0.90), caregiver engagement in employment status (OR = 0.81, 95% CI 0.73 to 0.91), and a medium or high level of caregiver awareness and knowledge of SMC (OR = 0.80, 95% CI 0.68 to 0.94; OR = 0.62, 95% CI 0.44 to 0.86), were all significantly associated with lower odds of spitting or vomiting. Directly supervised administration of Day 1 SPAQ was significantly associated with lower odds of spitting or vomiting (OR = 0.50, 95% CI 0.34 to 0.73).

**Table 3:** Results of a multilevel logistic regression model to predict children’s spitting or vomiting at the time of Day 1 SPAQ administration

Variable (n = 45,094)	OR	95% CI	*p*-value
Child			
Direct CD-supervised administration			
No	1.00		
Yes	0.50	0.34 to 0.73	<0.001***
Sex			
Female	1.00		
Male	0.78	0.64 to 0.95	0.012*
Age			
3–11 months	1.00		
1 year	1.41	1.08 to 1.83	0.011*
2 years	1.08	0.94 to 1.23	0.282
3 years	0.88	0.58 to 1.27	0.435
4 years	0.94	0.72 to 1.23	0.634
5 years	0.79	0.45 to 1.29	0.408
Primary caregiver			
Self-reported literacy			
No	1.00		
Yes	0.88	0.86 to 0.90	<0.001***
Level of highest education			
None or informal	1.00		
Primary	0.97	0.63 to 1.50	0.885
Secondary or higher	1.26	0.98 to 1.62	0.067
Employment status			
Non-employed	1.00		
Employed	0.81	0.73 to 0.91	<0.001***
Awareness and knowledge of SMC			
Low	1.00		
Medium	0.80	0.68 to 0.94	0.006**
High	0.62	0.44 to 0.86	0.005**
Head of household			
Employment status			
Non-employed	1.00		
Employed	0.94	0.73 to 1.20	0.599

^*^p < 0.05, **p < 0.01, ***p < 0.001

Model variables were selected using a forward stepwise procedure based on Collett’s method. OR calculated using random-effects multilevel logistic regression models for binary outcomes with random intercepts for survey year, country, and administrative subdivision (e.g., LGAs in Nigeria)

OR: odds ratio, CI: confidence interval, SMC: seasonal malaria chemoprevention, CD: community distributor

### Association between spitting or vomiting at the time of Day 1 SPAQ administration and adherence to AQ administration and caregiver-reported adverse reactions

Table 4 shows the results of chi-square tests for associations between spitting or vomiting at the time of Day 1 SPAQ administration, and adherence to Day 2 and Day 3 AQ administration, and caregiver reporting behavior following adverse reactions to any doses of SMC medicines by the selected child. Children experiencing spitting or vomiting were significantly less likely to receive Day 2 and Day 3 AQ from caregivers (*F* = 1498.86, df = 1, *p* < 0.001). Meanwhile, children who experienced spitting or vomiting were significantly more likely to have had adverse reactions to SMC medicines other than spitting or vomiting (*F* = 5.142, df = 1, *p* = 0.031) and their caregivers were less likely to report their adverse reactions to CDs or health facility personnel (*F* = 9.089, df = 1, *p* = 0.005).

**Table 4:** Associations between spitting or vomiting and other outcomes related to adverse reactions to SMC medicines

Variable	Spitting or vomiting	No spitting or vomiting	F-statistic	*p*-value^a^
	n (%)^b^	*n* (%)^b^		
Adherence to the second and third dose of AQ (*n* = 49,385)				
Yes	946 (78.58)	47,191 (97.75)	1498.86	<0.001***
No	237 (21.42)	1011 (2.25)		
Lead mothers visit (Nigeria only, *n* = 29,991)				
Yes	244 (45.1)	20,536 (69.58)	60.794	<0.001***
No	264 (54.9)	8947 (30.42)		
Adverse reactions to SMC medicines^c^ (*n* = 46,898)				
Yes	206 (19.31)	4641 (9.72)	5.142	0.031*
No	678 (80.69)	41,373 (90.28)		
Caregiver reporting of adverse reactions to CDs or health facility personnel^d^ (*n* = 4846)				
Yes	90 (46.18)	2960 (68.47)	9.086	0.005**
No	116 (53.82)	1680 (31.53)		

SMC: seasonal malaria chemoprevention, AQ: amodiaquine, CD: community distributor

^*^p < 0.05, **p < 0.01, ***p < 0.001

^a^Two-sided *p*-value for *χ*^2^ test for difference in proportions

^b^Weighted proportion based on weighted χ2 test

^c^Adverse reactions to the SMC medicines excluded “vomiting”

^d^Respondents who answered "yes" to "adverse reactions to the SMC medicines" were eligible to proceed to this question

### Characteristics of survey participants by redosing

Table 5 presents the results of weighted chi-square tests for associations between child-, caregiver-, head of household-, and household-level characteristics, and redosing of SMC medicines among children who spat or vomited the dose of Day 1 SPAQ. Children of primary caregivers who reported being literate (*F* = 18.14, df = 1, *p* < 0.001), who attained primary, secondary or higher education (*F* = 5.04, df = 2, *p* = 0.018), and who had a higher level of awareness and knowledge of SMC (*F* = 18.50, df = 2, *p* < 0.001), were significantly more likely to receive a new medicine dose.

**Table 5:** Summary of sample characteristics by redosing of SMC medicines to children experiencing spitting or vomiting

Variable (*n* = 1159)	Redosing	No redosing	F-statistic	*p*-value^a^
	*n* (%)^b^	*n* (%)^b^		
Child				
Direct CD-supervised administration				
Yes	369 (53.32)	409 (83.11)	13.64	<0.001***
No	299 (46.68)	82 (16.89)		
Sex				
Male	349 (47.32)	219 (42.57)	0.82	0.372
Female	319 (52.68)	272 (57.43)		
Age				
3–11 months	54 (8.56)	34 (5.72)	1.67	0.166
1 year	139 (22.24)	97 (22.11)		
2 years	175 (25.82)	108 (20.24)		
3 years	123 (17.25)	110 (22.23)		
4 years	136 (20.55)	112 (23.35)		
5 years	41 (5.58)	30 (6.35)		
Adverse reactions to SMC medicines^c^ (*N* = 864)				
Yes	93 (15.04)	108 (24.40)	5.579	0.025*
No	375 (84.96)	288 (75.60)		
Primary caregiver				
Sex				
Male	75 (9.65)	84 (16.40)	4.25	0.048*
Female	593 (90.35)	407 (83.60)		
Age				
Under 29 years	332 (49.35)	235 (46.54)	1.36	0.266
30–39 years	249 (36.86)	179 (36.57)		
40–49 years	62 (9.43)	61 (13.50)		
50 or more above	25 (4.36)	16 (3.40)		
Self-reported literacy				
Yes	328 (46.90)	311 (66.08)	18.14	<0.001***
No	340 (53.10)	180 (33.92)		
Level of highest education				
None or informal	388 (63.32)	240 (49.37)	5.04	0.018*
Primary	89 (12.08)	78 (15.03)		
Secondary or above	191 (24.60)	173 (35.60)		
Employment status				
Non-employed	289 (41.92)	210 (42.54)	0.01	0.929
Employed	379 (58.08)	281 (57.46)		
Awareness and knowledge of SMC				
Low	218 (36.23)	69 (15.82)	18.50	<0.001***
Medium	225 (36.40)	156 (33.05)		
High	225 (27.37)	266 (51.13)		
Caregiver reporting of adverse reactions to CDs or health facility personnel^d^ (*N* = 201)				
Yes	41 (48.72)	48 (45.56)	0.063	0.804
No	52 (51.28)	60 (54.44)		
Head of household				
Self-reported literacy				
Yes	385 (64.74)	323 (76.94)	7.72	0.010*
No	244 (35.26)	121 (23.06)		
Level of highest education				
None or informal	307 (54.34)	155 (35.48)	8.31	0.001**
Primary	71 (10.26)	66 (13.12)		
Secondary or above	228 (35.39)	212 (51.40)		
Employment status				
Non-employed	151 (19.76)	104 (22.45)	0.14	0.709
Employed	475 (80.24)	338 (77.55)		
Household				
Poverty				
Yes	83 (8.64)	89 (11.77)	1.27	0.268
No	585 (91.36)	402 (88.23)		
Lead mother visit (Nigeria only, *n* = 508)				
Yes	99 (32.58)	145 (62.58)	23.50	<0.001***
No	188 (67.42)	76 (37.42)		

SMC: seasonal malaria chemoprevention, AQ: amodiaquine, CD: community distributor

^*^p < 0.05, **p < 0.01, ***p < 0.001

^a^Two-sided *p* value for *χ*^2^ test for difference in proportions

^b^Weighted proportion based on corrected *χ*^2^ test

^c^Adverse reactions to the SMC medicines have excluded “vomiting”

^d^Respondents who answered "yes" to "adverse reactions to the SMC medicines" were eligible to proceed to this question

## Discussion

Recent updates to the WHO guidelines for SMC continue to emphasize the importance of appropriate dosing, and pharmacovigilance of adverse reactions to SMC medicines, in addition to potential piloting the use of alternative medicines such as dihydroartemisinin piperaquine (DP) which has been identified as an effective, well-tolerated artemisinin-based prevention strategy [5, 31]. It is advisable that eligible children with a history of adverse reactions after taking SP or AQ should not receive SMC; nevertheless, vomiting is not a contraindication [2]. Despite representing one of the most frequently reported adverse reactions associated with AQ, there has been a lack of investigation of spitting or vomiting and its associated sociodemographic factors under routine large-scale SMC implementation partly due to its lower severity and perceived implications for drug safety compared with other adverse reactions [16, 32].

Our analysis found a small proportion of children experienced spitting or vomiting at the time of Day 1 SPAQ administration in the four countries analyzed between 2020 and 2022, ranging from 1.81% in Nigeria to 4.36% in Chad. However, spitting or vomiting in the context of this study should be carefully interpreted as the eligible child spitting out most (almost all) of the dose or vomiting all the dose at the time of administration of Day 1 SPAQ. Spitting or vomiting in the context of our analysis did not include mild spitting as anecdotal evidence continues to suggest that mild spitting is very common during administration of SMC medicines.

The results of the regression analyses found that the children’s sex and age, caregivers’ literacy, employment status, and awareness and knowledge of SMC; and directly supervised administration of Day 1 SPAQ by CDs, were predictors of spitting or vomiting at the time of administration of Day 1 SPAQ. We found a strong association between caregiver awareness and knowledge of SMC and spitting or vomiting; the higher the level of knowledge of caregivers about the purpose, importance, and benefits of SMC and adherence to medications to their children in general, the less likely their children experience spitting or vomiting. This finding is consistent with those of both quantitative and qualitative studies which identified the critical role of knowledge of SMC in facilitating caregiver compliance to SMC implementation, by enhancing adherence to the complete course of SMC administration or allaying concerns regarding safety of medicines [14, 19, 33]. In addition, spitting or vomiting had a strong negative association with directly supervised administration of Day 1 SPAQ by CDs; this could be attributable to CDs’ training on advising caregivers on methods to administer medications and manage adverse reactions, and on answering questions from caregivers [4].

Furthermore, lower odds of adherence to Day 2 and Day 3 AQ administration and reporting of children's adverse reactions to CDs or health facility personnel were found among caregivers whose children experienced spitting or vomiting at the time of Day 1 SPAQ administration. This could be explained by caregivers considering children spitting or vomiting as uncommon adverse reactions and mistrusting the safety of further doses [33]. Ibinaiye et al. [19] and Cissé et al. [14] found previous adverse reactions to SMC medicines by eligible children (or perceived risk arising from administration of SMC medicines) were strongly associated with non-adherence to AQ administration. Caregiver non-reporting of children's adverse reactions may be also attributable to inadequate assistance and guidance from CDs.

Just under half of caregivers reported that their child did not receive a replacement dose following spitting or vomiting at the time of Day 1 SPAQ administration. Such low coverage of redosing was strongly associated with a low level of caregiver awareness and knowledge of SMC and a lack of directly supervised administration by CDs, consistent with a study by Ogbulafor et al. [33] that underscored the critical role of caregivers and CDs in ensuring compliance with SMC. However, it is difficult to determine to what extent each active or passive refusal by caregivers individually contributes to failure of redosing due to lack of availability of data on reasons for not receiving a replacement dose. Moreover, it is uncertain whether failure of redosing is due to CDs’ incomplete adherence to established procedures for SMC delivery, or other reasons such as stockouts of medicines.

## Strengths and limitations

Strengths of this study include its large, representative sample of households across multiple countries and years, providing high statistical power and wide generalizability of its findings. One limitation of this analysis is its high reliance on retrospective self-reporting by caregivers which may have resulted in potential social desirability bias and recall bias. Another limitation is that it is difficult to generalize the findings of spitting or vomiting at the time of Day 1 SPAQ administration to Day 2 and Day 3 AQ administration due to the unavailability of data.

Furthermore, there were no data on the presence of rapid diagnostic test-confirmed malaria cases after the final cycle of SMC administration. In addition, it was not possible to link health facility data on malaria cases to individual respondents, which may have facilitated analysis of causes of spitting or vomiting and failure of redosing.

## Policy implications

Actions taken to put these findings into practice have included sharing the study’s results with country program management and delivery teams, and then through cascade training to CDs. Greater emphasis may be placed on sensitization efforts by both CDs and at the community level. Particularly, direct messaging from CDs may also allay caregivers’ concerns around spitting or vomiting throughout the 3-day course of SMC administration, clarify the importance of full ingestion of SMC medicines and adherence to the 3-day course, and provide advice on how to prevent or ameliorate spitting or vomiting and on contacting the nearest point of care to obtain a replacement dose during unsupervised AQ administration on Day 2 and Day 3. Improving accessibility to health facilities and preventing stockouts of key commodities is also crucial. Furthermore, lead mothers visits and other country-tailored role model activities that have been proven to be effective in Nigeria and other countries to optimize SMC administration (e.g., encouragement tactics or discouragement of forced administration) may also be effective at reducing spitting or vomiting at the time of SMC administration [19].

## Conclusion

This study identified factors associated with spitting or vomiting, and redosing, of SMC medicines during household visits by CDs. Our analysis highlights the importance of direct CD-supervised administration of Day 1 SPAQ and caregiver awareness and knowledge of SMC in promoting full ingestion of SMC medicines. Continuing community sensitization activities and monitoring adverse reactions to SMC medicines remain essential.

## Data Availability

The data that support the findings of this study are available on reasonable request from the corresponding author SR.

## Abbreviations

AQ: Amodiaquine
CDs: Community distributors
CI: Confidence interval
COVID-19: Corona Virus Infectious Diseases-2019
LGAs: Local government areas
OR: Odds ratio
PPP: Purchasing power parity
SMC: Seasonal Malaria Chemoprevention
SP: Sulfadoxine-pyrimethamine
SPAQ: Sulfadoxine-pyrimethamine plus amodiaquine
USD: United States Dollars
WHO: World Health Organization
